# A Method for Optimal Detection of Lung Cancer Based on Deep Learning Optimized by Marine Predators Algorithm

**DOI:** 10.1155/2021/3694723

**Published:** 2021-08-11

**Authors:** Xinrong Lu, Y. A. Nanehkaran, Maryam Karimi Fard

**Affiliations:** ^1^Gannan University of Science & Technology, Ganzhou 341000, Jiangxi, China; ^2^Informatics School, Xiamen University, Xiamen 361005, Fujian, China; ^3^Non-Communicable Diseases Research Center, Rafsanjan University of Medical Sciences, Rafsanjan, Iran

## Abstract

Lung cancer is the uncontrolled growth of cells in the lung that are made up of two spongy organs located in the chest. These cells may penetrate outside the lungs in a process called metastasis and spread to tissues and organs in the body. In this paper, using image processing, deep learning, and metaheuristic, an optimal methodology is proposed for early detection of this cancer. Here, we design a new convolutional neural network for this purpose. Marine predators algorithm is also used for optimal arrangement and better network accuracy. The method finally applied to RIDER dataset, and the results are compared with some pretrained deep networks, including CNN ResNet-18, GoogLeNet, AlexNet, and VGG-19. Final results showed higher results of the proposed method toward the compared techniques. The results showed that the proposed MPA-based method with 93.4% accuracy, 98.4% sensitivity, and 97.1% specificity provides the highest efficiency with the least error (1.6) toward the other state of the art methods.

## 1. Introduction

Lung cancer is the uncontrolled and abnormal growth of cells that starts in one or two lungs. Abnormal cells do not grow in healthy tissues; they divide rapidly and form tumors. Primary lung cancer has its roots in the lungs, while secondary lung cancer begins elsewhere in the body, spreading from one part of the body to another and reaching the lungs [1]. The onset of lung cancer in the patient's body is indicated in most cases by early signs. The growing number of lung diseases in today's industrialized communities doubles the need for modern methods for accurate and early diagnosis.

Among lung diseases, lung cancer is still recognized as one of the most dangerous cancers. One-third of cancer deaths are due to lung cancer. About 80% of patients remain in the best condition for five years after being diagnosed with this type of cancer. Air pollution is one of the main causes of this disease. Early diagnosis of lung disease will have a major impact on the likelihood of definitive treatment of the disease. Major diagnostic methods for lung cancer include imaging, radiography and CT scan, biopsy, bronchoscopy, and examination of the breast mucosa. Pulmonary nodule is a small, round, opaque mass that forms inside the lung tissue. In other words, nodules are spherical radiographic opacities less than 30 mm in diameter. So far, various researches have been done in order to identify and describe lung diseases. Due to the removal and high number of radiographic images of the lungs, as well as its complex and uneven structure, it is difficult for a specialist physician to diagnose nodules from vessels, wounds, etc. A computer diagnostic aid system is a system that assists a physician in diagnosing a disease. Such systems are used as an intelligent tool that expresses a second option to the radiologist, which shows suspicious situations in the images to the radiologist and thus helps the radiologist to make the most accurate diagnosis. The basic idea is not to leave the diagnosis to a machine, but to use it because it increases the sensitivity of the work and reduces the rate of positive error. Several works were introduced in this subject.

Hussain et al. [2] presented a method based on multiscale sample entropy (MSE) with a mean and KD-tree algorithmic approach, multiscale fuzzy entropy (MFE), refined composite multiscale fuzzy entropy (RCMFE), and multiscale permutation entropy (MPE) for the diagnosis of the lung cancer. The results showed that the MFE-based texture features with standard deviation provide the highest accuracy with 1.95*E* − 50 *P* value. Simulation results indicated that the developed measures using RCMFE outdone the others in dynamics analysis of the lung cancer.

Lakshmanaprabu et al. [3] proposed an automatic detection system for the classification of lung cancer in computed tomography (CT) images of lungs. They analyzed the CT scan of lung images based on linear discriminate analysis (LDA) and optimal deep neural network (ODNN). Features that were extracted from the CT lung images were then reduced by an LDR to reduce the features dimension. The system was a binary classification to show if the data is benign or malignant. The ODNN was then performed to CT images and optimized by a modified gravitational search algorithm (MGSA) to provide a method with higher accuracy.

Wang et al. [4] introduced a weakly supervised technique for efficient and fast diagnosis of the lung cancer from the images. They first used a patch-based fully convolutional network (FCN) to save discriminative blocks and offer illustrative deep features with high efficiency. Afterward, diverse feature aggregation and context-aware block selection policies were used to make globally holistic WSI descriptor. Then, it fed into a random forest classifier. Final results showed the system efficiency.

Shakeel et al. [5] proposed another technique for the diagnosis of the lung cancer. They provided a developed diagnosis system based on misclassification reduction. Weighted mean histogram equalization was used for noise elimination of the input images, and improved profuse clustering technique (IPCT) was used for images quality enhancement. Spectral features were extracted from the region of the interest, and then, deep learning was used for lung cancer prediction. Simulation results have some advantages and disadvantages that showed the proper efficiency of the proposed method. As can be observed from the literature, several works are proposed for efficient diagnosis of the lung cancer. However, using each of them has its advantages and disadvantages. In this study, we attempt to use deep learning technique and metaheuristic technique for providing a method with higher efficiency for the diagnosis of the lung cancer.

The main contributions of the present study are highlighted as follows:

1. Presenting a new method for lung cancer diagnosis from the lung CT scan images
2. Proposing a new structure for convolutional neural network as an efficient tool for the cancer diagnosis
3. Optimizing the convolutional neural network based on a newly introduced metaheuristic, called marine predators algorithm

## 2. Methodology

**Figure 1:**
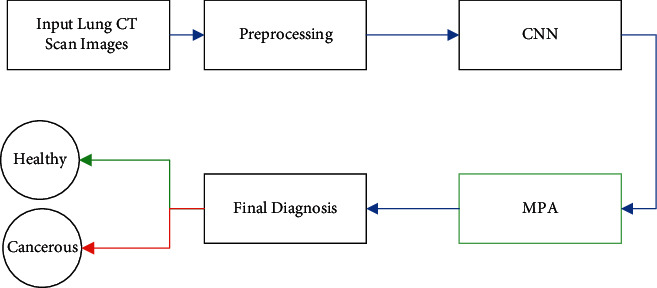
The graphical abstract of the proposed method.

Recently, convolutional neural networks (CNNs) have been turned into one of the most popular technologies in the medical imaging technology. Most of the application of deep learning in the cancer screening is based on CNNs. Convolutional neural network is a deep learning algorithm that receives the input image and assigns importance (learnable weights and biases) to each of the objects/aspects in the image and is able to distinguish them from each other. The CNN algorithm requires less “preprocessing” than other classification algorithms. While the primary method filters are manually engineered, the CNN acquires the ability to learn these filters/specifications with sufficient training [6]. The CNN architecture is similar to the connection pattern of “neurons” in the human brain and is inspired by the organization of the “visual cortex” in the brain. Each neuron responds to stimuli only in a limited area of the visual field known as the “Receptive Field.” A set of these fields overlap to cover the entire visual area. In this research, the application of convolutional neural network on the lung cancer diagnosis has been investigated. To determine the cancerous or healthiness of this disease, we used deep neural networks based on MATLAB software. The graphical abstract of the proposed method is given in Figure 1.

### 2.1. Preprocessing Step

The first stage in the proposed method is to preprocess the input data to remove all cases that can imply bad effects on the CNN. The preprocessed images are then injected to a CNN with specific architecture and training, and test on the image data begins. The lung images dataset even with the best imaging contain some lights and noises which should be eliminated. These noises may even affect the precision of the final classifier. Pixels with high frequencies have high destructive effects on the images which can be removed with adding a low-pass filter.

#### 2.1.1. Noise Removal

**Figure 2:**
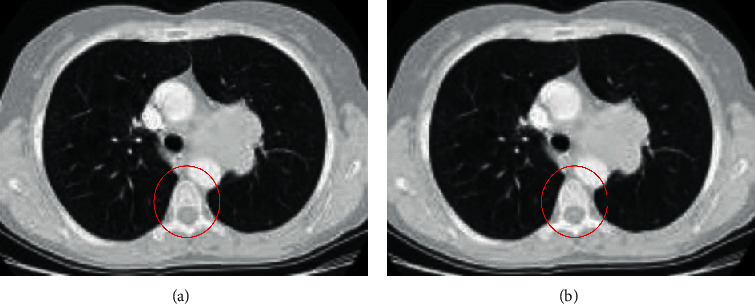
A simple example of performing the noise removal on the lung images based on median filtering.

As mentioned before, in medical imaging, noise removal from the input images is a noteworthy task. Using noise removal stage should be done by leaving the images edges unchanged and keeping the image sharpness as much as possible. Median filtering is a popular low-pass filter such that each output pixel is established from the average brightness values of the neighboring pixels based on the input pixels [7]. In median filtering, the amount of a pixel has been achieved by the middle amount of the neighboring pixels. Since the center filter has low sensitivity to throw values, it can eliminate these points with no image resolution decreasing. This filter also decreases the light intensity variance, while keeping the shape of the edges and their position [8]. This filter by *m* ×  *n* neighborhood sorts in ascending order, with central element selection of the sorted values and replacing by the center pixels. The median filter also can remove the salt and pepper noises easily [9]. Therefore, in this study, we utilized this filter as a preprocessing stage of the input images. In median filtering, pixels are replaced by the median value of their neighbors, i.e.,

(1) $$\begin{array}{c} y_{\left(m,n\right)}=\text{median}\left(x_{i,j}:\left(i,j\right)\in \tau \right), \end{array}$$

where *τ* defines the nearby neighbors in (*m*, *n*).  Figure 2 shows a simple example of performing the noise removal on the lung images based on median filtering. It should be noted that the size of filter in this example is 5 × 5.

As can be observed from Figure 1, the method provides a small filtering on the image, while it keeps the other details.

#### 2.1.2. Image Level Balancing

After noise removing, normalization is required to scale the acquired images between 0 and 1 for simplifying the complexity of the dataset. Here, we used min-max normalization method. The present study employs 250 × 250 scale normalization for this problem. With assuming a grayscale image with *n* dimension and the following limitation, i.e., *I*_*n*_=[*X*⊆*R*^*n*^]⟶[*a*,…, *b*], the normalized image, *I*^*∗*^, is formulated as follows:

(2) $$\begin{array}{c} I^{*}=a_{\text{new}}+\frac{b_{\text{new}}-a_{\text{new}}}{b-a}\times \left(I-a\right), \end{array}$$

where *a* and *b* represent the intensity values of the image, *I*^*∗*^=[*X*⊆*R*^*n*^]⟶[*a*_new_,…, *b*_new_], and *a*_new_ and *b*_new_ signify the intensity values for the normalized image [10].

### 2.2. Convolutional Neural Network

**Figure 3:**
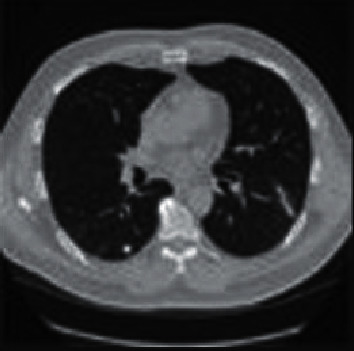
An example of preprocessed image.

In the architecture of this research, some images with the same scale are needed for processing. Therefore, all of the images have been rescaled to 227 × 227 before training by the CNN. In Figure 3, a simple example of this method on the considered database is shown.

The network training is indeed minimizing the error function between the network real outputs and the network desired outputs. This is done by improving the free parameters of the network, i.e., weights and biases [11]. The method of training in this study is supervised. In this method, a supervisor handles the training behavior and trains the network the correct way of learning. In other words, some examples of inputs and outputs are presented to the network [12]. Afterward, the output of the network is compared with the desired output to achieve the amount of the error value. Then, the weights and biases are selected to minimize this value. The training of the network parameters can be established based on two ways: training after each training sample which is called “sequential,” or the training updated after applying all of the training samples which is called “batch.”

**Figure 4:**
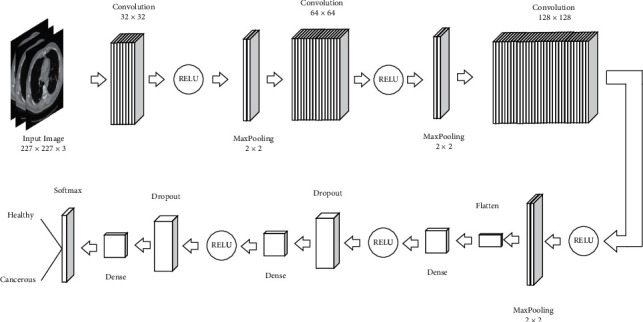
The proposed CNN model for the studied case.

The first method needs less memory, but, it has less stability because each training sample can address the network parameters into a new direction. In the second method, although it needs more memory for storing the parameters, it will have more stability. Therefore, in the following, we used the batch mode. In this study, we use batches with scale 32 to train the database images. In the deep neural network's architecture, 3 numbers of “convolutional” layers and three numbers of “max pooling” layers have been used.  Figure 4 shows the total architecture.

The convolutional layer contains the main kernel of the CNN, and output mass can be as a 3D mass of neurons. Convolution is a mathematical operation for the process of the signal that is utilized for applying the convolutional operations to the inputs by the neurons. The most important parameter in the convolutional layers is their filter size where, in the proposed model, the three layers of 32 × 32, 64 × 64, and 128 × 128 have been used. After the convolutional layer, to reduce the special size (depth), pooling layer has been utilized. This helps to decrease the parameters numbers and to increase the network speed. The pooling layers reduce the number of the output layers of the filter. In this study, we used 2 × 2 filter for this purpose. Indeed, the main idea is to subsample the input image to reduce the complexity cost, memory, and the number of parameters in the network.

**Figure 5:**
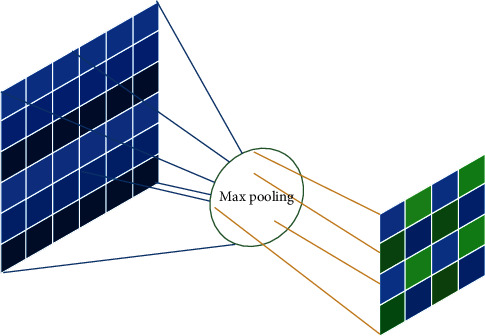
The method of max pooling.

The size reduction of the input image also decreases the sensitivity of the neural network. In the pooling layer, like convolutional layer, each neuron is connected to the output of some neurons. To reduce the size and increase the computation speed, pooling layer for sampling is used. In this research, the pooling layer with a 2 × 2 window steps on the image. From the four existing pixels in this window, the maximum pixel is selected and transferred to the next layer. The method of max pooling is shown in Figure 5.

**Figure 6:**
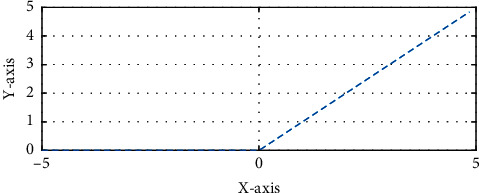
The performance profile of the RELU activation function.

After each convolutional layer, an activation layer is used where its target is to introduce nonlinear operations to a system that completely computed the linear operations in the computational layers [13]. This research uses RELU layers for this purpose because the network due to the computational efficiency can train faster without changing in the accuracy. The performance profile of the RELU activation function is shown in Figure 6. The RELU layer applies the function *f*(*x*) to all of the input images and converts all of the negative activations into the zero. With using this layer, the nonlinear features of the model and the network have been increased with no effect on the convolutional layers.

**Figure 7:**
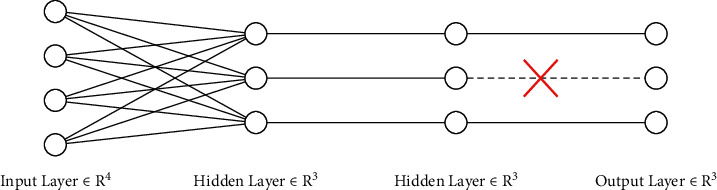
The method of performing the dropout layer.

By considering the “dropout” layer during the training, based on a definite probability, the output of some neurons has become zero where, by this operation, a different network is accessible, and this network when facing with independence to other neurons discovers and uses strong features. Therefore, the dropout technique is used to prevent the overfitting problem [14]. Because the parameters of a fully-connected deep network are a lot, the training will be slow, and during the training step, the probability for premature convergence has been increased. Therefore, among the fully-connected layers, the dropout layer has been used for the parameter's reduction.  Figure 7 shows the method of performing the dropout layer.

After convolutional and pooling layers, lots of feature data with low sizes will have been produced. With connecting these layers to a Softmax classifier, due to the fact that the input images marks in training stage are determined, the network can be trained for classification of all images by entering all of the trained images and their marks. During the training process, the system is looking for the best unknown parameters, especially filter weights and the layers coefficients such that the minimum error value has been achieved for the classification. With considering the output “Flatten” layer, the convolutional layers that are a multidimensional tensor converted to a 1D tensor, and finally using the “Optimizer RMSprop” that is used for evaluating several activation functions in tensor flow, the weights optimization has been done. In the test stage, the remained images from the data that are not used for training utilized for test the network. In this condition, the output of the layers is used as the demanded image feature vector. At last, the image feature vector is compared with the feature matrix, and the deep neural network uses some layers for understanding some parts of data; however, for the classification of data, we should have a collect of probabilities for the final decision [15]. Softmax is a popular function that is used for normalizing the probability values in a standard range (0 to 1).

As aforementioned, “Optimizer RMSprop” method has been done for optimal determining of the weights. The optimization operation in the system is based on minimizing the cross-entropy [16] which can be mathematically formulated as follows:

(3) $$\begin{array}{c} L=\sum_{j=1}^{N}\sum_{i=1}^{M}-d_{j}^{i}\log\;z_{j}^{i}, \end{array}$$

where *N* defines the samples number, $d_{j}=\left(0,\ldots ,0,\underset{k}{\underset{︸}{1,\ldots ,1}},0,\ldots ,0\right)$ signifies the desired output vector, and *z*_*j*_ represents the obtained output vector of the *m*^th^ class which is formulated below:

(4) $$\begin{array}{c} z_{j}^{\left(i\right)}=\frac{e^{f_{j}}}{\sum_{i=1}^{M}e^{f_{i}}}. \end{array}$$

Equation (3) can be extended by considering a weight penalty as *η* as follows:

(5) $$\begin{array}{c} L=\sum_{j=1}^{N}\sum_{i=1}^{M}-d_{j}^{i}\log\;z_{j}^{i}+\frac{1}{2}\eta \sum_{K}\sum_{O}\omega_{k,l}^{2}, \end{array}$$

where *O* signifies the total number of layers, *K* describes the connections in layer *l*, and *ω*_*k*_ defines the connection weight.

Numerous research works are presented for optimal arrangement of the CNNs [17]. In the present research, we also work on a new optimal technique based on metaheuristic for this purpose.

In this study, we first determine the minimum (min) and the maximum (max) limitations of the algorithm to avoid the system errors, such that min is set 2 as the minimum value that is acceptable for max pooling, and max defines the size of the sliding window. It should be noted that the input data value should be larger than the sliding window. The initial population is set 100, and the hyperparameters settings of the CNN are described 10 integer values. Then, the solutions are assessed. In this study, we used the half-value precision to the optimized CNN as the cost function. It should be noted that the overall methodology needs high computational cost because all of the population members need to be trained on the lung cancer dataset with performing backpropagation algorithm. In this study, two features are selected for optimizing as follows:

(6) $$\begin{array}{c} W=\left\{w_{1},w_{2},\ldots ,w_{p}\right\},\\ A=\left\{a_{1},a_{2},\ldots ,a_{A}\right\},\\ w_{n}=\left\{w_{1n},w_{2n},\ldots ,w_{Ln}\right\},\\ b_{n}=\left\{b_{1n},b_{2n},\ldots ,b_{Ln}\right\},\;l=1,2,\ldots ,Ln=1,2,\ldots ,A, \end{array}$$

where *l* defines the layer index, *n* signifies the number of agents, *w*_*in*_ describes the value of the weight in layer *i*, and *L* and *A* represent the total number of layers and the total number of agents, respectively. The error cost function for the CNN is provided as follows:

(7) $$\begin{array}{c} E=\frac{1}{T}\sum_{i=1}^{T}\sum_{j=1}^{k}{\left(d_{ji}-o_{ji}\right)}^{2}, \end{array}$$

where *k* describes the number of output layers, *T* signifies the number of training samples, and *d*_*ji*_ and *o*_*ji*_ represent the desired and the output value of the CNN. This method has been easily trapped into the local minimum that can be resolved by using the metaheuristics. In addition, metaheuristics do not need backward phase as a high computational cost process. In this study, marine predators algorithm has been used for this purpose which is explained in the following section.

### 2.3. Marine Predators Algorithm

In 2020, Faramarzi et al. proposed a new metaheuristic algorithm, called marine predators algorithm (MPA) for solving the optimization problems [18]. During the MPA, to model the relation between prey and marine predators, the predators utilize foraging methodology and Lévy random movement and Brownian behavior. If the attraction sense for the prey in the hunting region is high, predators employ Brownian method; if not so, if attraction sense of prey is low, Lévy method has been used. Ecological problems such as fish aggregating devices (FADs) effects or eddy formation alternates the marine predator's behavior [19]. In MPA, at first, initial *n* numbers of population for the prey are defined with considering a lower and an upper bound, i.e.,

(8) $$\begin{array}{c} {\overset{⟶}{X}}_{i}={\overset{⟶}{X}}_{\min}+\overset{⟶}{\text{rand}}\left(0,1\right)⊗\left({\overset{⟶}{X}}_{\max}-{\overset{⟶}{X}}_{\min}\right),\;i=1,\ldots ,n, \end{array}$$

where rand defines d-dimensional random values that are uniformly distributed in the range between 0 and 1, ${\overset{⟶}{X}}_{i}$ signifies the *i*^th^ candidate in the initial population for prey, and ${\overset{⟶}{X}}_{\min}$ and ${\overset{⟶}{X}}_{\max}$ describe the d-dimensional vectors of the lower and the upper bounds for the search space.

Afterward, the cost value for the candidates has been measured based on cost function. Based on the survival of the fittest mechanism, the candidate which has the best cost value has been selected as the top candidate for the solution. Lastly, the top member vector has been replicated *n* times to achieve the elite matrix as follows:

(9) $$\begin{array}{c} \text{Elite}=\left[\begin{array}{ccc} \begin{array}{cc} \begin{array}{c} X_{1,1}^{B}\\ X_{2,1}^{B} \end{array} & \begin{array}{c} X_{1,2}^{B}\\ X_{2,2}^{B} \end{array} \end{array} & \cdots & \begin{array}{c} X_{1,d}^{B}\\ X_{2,d}^{B} \end{array}\\ \vdots & \ddots & \vdots \\ \begin{array}{cc} X_{n,1}^{B} & X_{n,2}^{B} \end{array} & \cdots & X_{n,d}^{B} \end{array}\right], \end{array}$$

where *n* describes the population size, *d* describes the problem dimension, and $\overset{⟶}{X^{B}}$ describes the vector for the top member of the predator population. The prey candidates vector is defined as follows:

(10) $$\begin{array}{c} \text{prey}=\left[\begin{array}{ccc} \begin{array}{cc} \begin{array}{c} X_{1,1}\\ X_{2,1} \end{array} & \begin{array}{c} X_{1,2}\\ X_{2,2} \end{array} \end{array} & \cdots & \begin{array}{c} X_{1,d}\\ X_{2,d} \end{array}\\ \vdots & \ddots & \vdots \\ \begin{array}{cc} X_{n,1} & X_{n,2} \end{array} & \cdots & X_{n,d} \end{array}\right], \end{array}$$

where *X*_*i*,*j*_ describes the *j*^th^ dimension for the *i*^th^ prey.

It should be noted that elite and prey matrices contain two significant characteristics of the MPA. The foraging process in the MPA has been divided into three main portions that are described in the following. The first part is high-velocity ratio. Here, this mechanism simulates the exploration term of the algorithm and resembles the time when the prey moves quicker than the predator. The mathematical model of the exploration phase for MPA is as follows.

If Iteration < (1/3)*∗*Max_Iteration,

(11) $$\begin{array}{c} {\overset{⟶}{\text{Step}}}_{i}={\overset{⟶}{R}}_{B}⊗\left({\overset{⟶}{\text{Elite}}}_{i}-{\overset{⟶}{R}}_{B}⊗{\overset{⟶}{\text{Prey}}}_{i}\right),\\ {\overset{⟶}{\text{Prey}}}_{i}={\overset{⟶}{\text{Prey}}}_{i}+P*\overset{⟶}{R}⊗{\overset{⟶}{\text{Step}}}_{i}, \end{array}$$

where ${\overset{⟶}{R}}_{B}$ describes d-dimensional vector including some random values based on Brownian motion, the operator ⊗ signifies the entry-wise multiplication, *R* describes a uniformly distributed value in the range [0, 1], and *P* is a constant value equal to 0.5.

The second part contains unit velocity ratio. In this part, both prey and predator move equally. This is the intermediate part of the optimization and contains both exploitation and exploration stages where the exploration phase regularly becomes the exploitation phase. Here, the population has been divided into two portions. Half of it has been utilized for exploration, and the other half has been utilized for exploitation. If (1/3) × Max_Iteration < Iteration < (2/3) × Max_Iteration, for the first half, we have

(12) $$\begin{array}{c} {\overset{⟶}{\text{Step}}}_{i}={\overset{⟶}{R}}_{L}⊗\left({\overset{⟶}{\text{Elite}}}_{i}-{\overset{⟶}{R}}_{L}⊗{\overset{⟶}{\text{Prey}}}_{i}\right),\\ {\overset{⟶}{\text{Prey}}}_{i}={\overset{⟶}{\text{Prey}}}_{i}+P*\overset{⟶}{R}⊗{\overset{⟶}{\text{Step}}}_{i}. \end{array}$$

To the other population half candidates,

(13) $$\begin{array}{c} {\overset{⟶}{\text{Step}}}_{i}={\overset{⟶}{R}}_{B}⊗\left({\overset{⟶}{R}}_{B}⊗{\overset{⟶}{\text{Elite}}}_{i}-{\overset{⟶}{\text{Prey}}}_{i}\right),\\ {\overset{⟶}{\text{Prey}}}_{i}={\overset{⟶}{\text{Elite}}}_{i}+P*CF⊗{\overset{⟶}{\text{Step}}}_{i}, \end{array}$$

where *R*_*L*_ signifies a random vector based on Levy-Flight (Lévy motion) and *CF* represents an adaptive parameter to control the predator motion step as follows:

(14) $$\begin{array}{c} CF={\left(1-\frac{\text{Iteration}}{\text{Max}\_\text{Iteration}}\right)}^{\left(2*\left(\text{Iteration}/\text{Max}\_\text{Iteration}\right)\right)}. \end{array}$$

The third part includes low-velocity ratio which is accomplished when the prey moves with lower speed than the predators and the exploitation stage is finished. If Iteration > (2/3) × Max_Iteration,

(15) $$\begin{array}{c} {\overset{⟶}{\text{Step}}}_{i}={\overset{⟶}{R}}_{L}⊗\left({\overset{⟶}{R}}_{L}⊗{\overset{⟶}{\text{Elite}}}_{i}-{\overset{⟶}{\text{Prey}}}_{i}\right), \end{array}$$

and in MPA, marine predators spend 80% of their time looking for prey and the remaining 20% looking for more extensively for a diverse environment with various prey distribution. This is modeled by the following equation:

(16) $$\begin{array}{c} {\overset{⟶}{\text{Prey}}}_{i}=\left\{\begin{array}{c} {\overset{⟶}{\text{Prey}}}_{i}+CF*\left[{\overset{⟶}{X}}_{\min}+\overset{⟶}{R}⊗\left({\overset{⟶}{X}}_{\max}-{\overset{⟶}{X}}_{\min}\right)\right]⊗\overset{⟶}{U}ifr\;\leq \text{FADs},\\ {\overset{⟶}{\text{Prey}}}_{i}+\left[\text{FADs}*\left(1-r\right)+r\right]*\left({\overset{⟶}{\text{Prey}}}_{r1}-{\overset{⟶}{\text{Prey}}}_{r2}\right)ifr>\text{FADs}, \end{array}\right. \end{array}$$

where $\overset{⟶}{U}$ describes a binary vector with initial value equal to zero and FADs are considered 0.2, *r* describes a random value between 0 and 1, and *r*_1_ and *r*_2_ define two members of the prey population which are selected randomly.

## 3. Dataset Description

**Figure 8:**
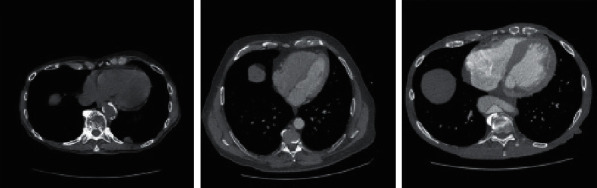
Some samples of RIDER Lung CT.

Since the diagnosis capability analysis is so important before using it as a tool, the method efficiency should be evaluated by applying it to a dataset. In this study, the method is tested based on the Reference Image Database to Evaluate Therapy Response (RIDER) dataset [20]. This project was first originated in 2004 as a collaboration among the NCI's Center for Bioinformatics, the Cancer Research and Prevention Foundation (CRPF), the NCI's Cancer Imaging Program, and the National Institute of Biomedical Imaging and Bioengineering (NIBIB) with information technology support from the Radiological Society of North America (RSNA). The RIDER dataset has been downloaded from the Cancer Imaging Archive [20]. The RIDER Lung CT Collection includes images from repeated lung CT scans (“coffee break experiment”) for 32 subjects detected with non-small-cell lung cancer. The collection also comprises a spreadsheet of recognized lesions where each of them experienced 2 CT studies on the same scanner and the same imaging protocol. In Figure 8, some samples of RIDER Lung CT are shown.

## 4. Evaluation Criteria

**Figure 9:**
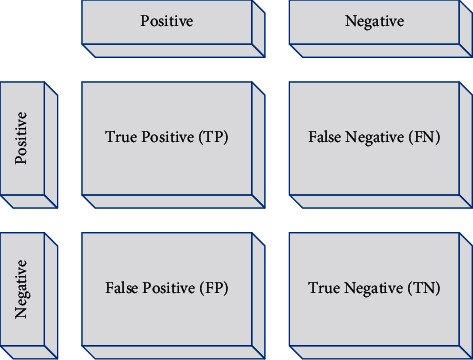
The matrix of cluttered elements.

The accuracy measurement indicator is a criterion that assesses the capability of the model in the relation of the results with the existing information features.  Figure 9 shows the matrix of cluttered elements.

Equation (17) shows the accuracy criteria formulation:

(17) $$\begin{array}{c} \text{accuracy}=\frac{\text{TN}+\text{TP}}{\text{TN}+\text{FN}+\text{TP}+\text{FP}}. \end{array}$$

In statistics, there are two accuracy and correctness for analyzing the result of the classification method; in other words, when we can divide the data into two groups of positive and negative, the accuracy of the results of a test which divide the information into these two classes can be measurable and definable by the sensitivity analysis [21]. The sensitivity is a ratio of the positive cases that experiment marks them correctly as positive cases. The sensitivity of a test depends only on the test nature and the tested sample. Nevertheless, by only using the sensitivity, we cannot interpret a test. The equation of the sensitivity is given as follows:

(18) $$\begin{array}{c} \text{sensitivity}\left(\text{recall}\right)=\frac{\text{TP}}{\text{TP}+\text{FP}}. \end{array}$$

The classification error criteria are completely opposite of its classification accuracy criteria which are obtained by the following formula:

(19) $$\begin{array}{c} \text{error}=\frac{\text{FN}+\text{FP}}{\text{TN}+\text{FN}+\text{TP}+\text{FP}}, \end{array}$$

where its minimum value is zero, i.e., when the maximum and the best performance have been achieved.

## 5. Results

The deep training tools for the identification of the key features from the complicated dataset indicate the importance of them in this modern medical century. In the proposed method in this study, 80% of the images from the dataset are randomly selected for training and 20% of remaining images are used for test, while there is no interconnection between the training and test data.

The training data with batch size 32 are sent to the deep neural network, where this network is trained during 200 iterations. The proposed model by using the sequential training technique and extracting the high-level features provides better performance than the other methods.  Table 1 indicates the simulation results of the proposed method and its comparison with others.

**Figure 10:**
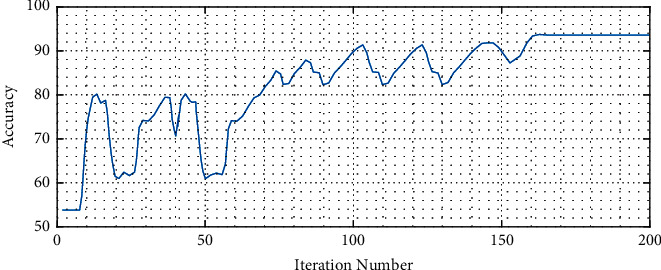
The convergence profile of the accuracy for the diagnosis of the cancer.

The results from Table 1 show that the proposed method has higher accuracy than the other compared methods.  Figure 10 shows the convergence profile of the accuracy for the diagnosis of the cancer.

As can be observed, the proposed method is converged in iteration 166.

## 6. Discussions

Recently, deep learning is widely used in different fields of machine vision, such as image classification, object detection, and segmentation. The images classification can be also accurately performed by using deep neural networks on the databases. There are several works about automatic detection of cancer in machine vision and deep learning. These research attempts provide worthy results in this area. In this study, we utilized a designed convolutional neural network for the lung cancer detection. The results of the proposed method are compared with some other pretrained CNN ResNet-18, GoogLeNet, AlexNet, and VGG-19. The proposed method has been trained based on two methods: classic (Optimizer RMSprop) method and a new metaheuristic-based (MPA) method. The results showed that the proposed method based on MPA with 93.4% accuracy has the highest accuracy value.

## 7. Conclusions

Due to the reduction of the living standards such as sedentary lifestyles, poor eating habits, and increased smoking, cancer rates have risen dramatically in the last century, prompting researchers and scientists to take steps to treat this dangerous disease. Research by scientists has shown that the early detection of this disease makes it easier to treat, and the risk of death from this disease is reduced. In this article, we will discuss a new method for early detection of lung cancer. Here, an automatic system based on deep neural networks was proposed for optimal diagnosis of the CT-based lung images, in medical images. The proposed method used a metaheuristic technique, called marine predators algorithm for optimal arrangement and better network accuracy. Due to the extraction of the high-level features based on deep networks in the proposed method, the classification and diagnosis accuracy were high. Moreover, the feature vector size was reduced, and in the initial steps, its accuracy was increased that accordingly reduced the saving space and increased the speed and accuracy. In the future work, we study modifying the proposed method to provide the system with higher speed to be feasible in the real-time application.

## Figures and Tables

**Table 1:** The simulation results of the proposed method and its comparison with others.

Method	Accuracy	Sensitivity	Specificity	Error
Proposed method/MPA	93.4	98.4	97.1	1.6
Optimizer RMSprop/optimizer RMSprop	88.0	92.9	88.3	12.0
ResNet-18	89.3	93.4	68.5	6.6
GoogLeNet	68.2	71.5	48.2	31.8
AlexNet	88.6	92.7	72.7	11.4
VGG-19	82.7	91.6	73.6	17.3

## Data Availability

The data that support the findings of this study are available at https://wiki.cancerimagingarchive.net/display/Public/RIDER+Lung+CT.
